# Supplementary material for: Azhdarchid pterosaur diversity in the Bayanshiree Formation, Upper Cretaceous of the Gobi Desert, Mongolia
Source: PeerJ. 2025 Sep 16;13:e19711. doi: 10.7717/peerj.19711 (PMC12447946; doi:10.7717/peerj.19711)
Supplement: Supplemental Information 2 [file peerj-13-19711-s002.docx]

**SUPPLEMENTAL TEXT**

**Azhdarchid pterosaur diversity in the Bayanshiree Formation, Upper Cretaceous of the Gobi Desert, Mongolia**

Rodrigo V. Pêgas, Xuanyu Zhou, and Yoshitsugu Kobayashi

1. ***Wingspan estimates***

As mentioned in the main text, estimating body size for fragmentary fossil specimens is a complicated task, particularly when it comes to groups with large variations in skeletal proportions – such as azhdarchids (e.g. Cai & Wei, 1994; Vremir *et al.*, 2015; Andres & Langston, 2021). The herein described Baynshiree Formation azhdarchids are represented solely by cervical vertebrae. We note here that mesocervical vertebrae length cannot be reliably utilized for making body size comparisons within azhdarchids, due to the great variation in mesocervical relative length in this group, wherein some forms are “slender-necked” while others are more “robust-necked” (see Naish & Witton, 2017; Pêgas *et al.*, 2021). Of note, cervical length/humerus length (CL/HL) ratios vary quite extremely across azhdarchids. Regarding cervical IV, CL/HL ratios are 1.09 for *Q. lawsoni* (mean value; Andres & Langston, 2021), 0.85 for *Zhejiangopterus linhaiensis* (Cai & Wei, 1994), and 0.56 for *Cryodrakon boreas* (Hone *et al.*, 2019). The fourth cervical of *Q. lawsoni* is almost twice as elongate relative to the humerus if compared to *Cryodrakon boreas*. Despite this tremendous difference in cIV relative elongation, their prezygapophyseal width/humeral length ratios are quite similar: 0.29 for *Q. lawsoni* (mean value; Andres & Langston, 2021) and 0.26 for *Cryodrakon boreas* (Hone *et al.*, 2019). Therefore, it seems that width proportions are more conservative than length proportions in azhdarchid cervicals. For this reason, the present work will focus in cervical width, rather than length, ratios.

Ideally, body size estimates should be produced by regression analyses to account for allometric corrections. Unfortunately, relatively complete azhdarchid skeletons are exceedingly rare, and restricted to the taxa *Zhejiangopterus linhaiensis* (Cai & Wei, 1994), *Mistralazhdarcho maggi* (Vullo *et al.*, 2018), *Cryodrakon boreas* (Hone *et al.*, 2019), and *Quetzalcoatlus lawsoni* (Andres & Langston, 2021). Performing regression and allometric analyses with such a low sample is not feasible. Still, multiple comparisons (using atlantoaxis proportions in *Mistralazhdarcho maggi* and *Quetzalcoatlus lawsoni*, as well as width in the third and sixth cervicals) converge towards a wingspan of 3.0–3.5 for MPC−Nd 100/302, as explored below.

Firstly, the estimated wingspan of *Mistralazhdarcho maggi* needs to be addressed. The holotype of *Mistralazhdarcho maggi* preserves a radius (the only completely preserved forelimb element) of 289 mm in length, which is ~124% and ~76% the radius lengths of, respectively, the ~3 meter-wingspan *Zhejiangopterus linhaiensis* specimen ZMNH M 1323 (see Cai & Wei, 1994) and the ~4.8 meter-wingspan *Quetzalcoatlus lawsoni* specimen TMM 42138-1 (Andres & Langston, 2021). These proportions suggest, respectively, a wingspan between 3.6 and 3.7 m for the holotype of *Mistralazhdarcho maggi*. It is interesting to note that the atlantoaxes of specimen MPC−Nd 100/302 and of the holotype of *Mistralazhdarcho maggi* are almost exactly of the same size (centrum lengths x condyle widths of, respectively, 26 x 19.8 mm and 26 x 20 mm); thus indicating they most likely represent similar-sized individuals.

Comparisons between the atlantoaxes and third cervicals of MPC−Nd 100/302 and *Quetzalcoatlus lawsoni* may also be informative, though less useful given that the available atlantoaxes and third cervicals of *Q. lawsoni* represent isolated specimens of unknown wingspan. Still, considering that the *Q. lawsoni* bone bed comprises specimens between 4.2–5.1 m in wingspan (see forelimb measurements in Andres & Langston, 2021), estimates can be tentatively drawn on the basis of this range. Atlantoaxis condyle width in *Q. lawsoni* specimen TMM 41954-36 (24 mm; Andres & Langston, 2021) correspond to 120% of the same value in MPC−Nd 100/302, thus suggesting a wingspan range of 3.5–4.2 m for the latter specimen. Similarly, cervical III prezygapophyseal and postzygapophyseal widths in *Q. lawsoni* specimen TMM 42422-24 (50 and 48.8 mm; Andres & Langston, 2021) correspond to 130% and 127% of the same value in MPC−Nd 100/302, thus suggesting a wingspan range of 3.2–4.0 m for the latter specimen. The lower values (3.2–3.5) seem most plausible than the higher ones, given that the aforementioned *Q. lawsoni* specimens are not among the largest ones, even though their precise wingspan cannot be assessed (Andres & Langston, 2021). Furthermore, CVI condylar width in MPC−Nd 100/302 (24.3 mm) is ~69% this value in *Q. lawsoni* (35 mm), what would suggest a wingspan of 2.9–3.5 for MPC−Nd 100/302.

Finally, it must also be noted that the CIII of MPC−Nd 100/302 (38.5 mm in prezygapophyseal width) is only slightly larger than that of the holotype of *Eurazhdarcho langendorfensis* (37 mm in prezygapophyseal width), which is estimated at 3 m wingspan based on its wing remains (Vremir *et al.*, 2013). Therefore, taking all of the abovementioned evidence together, a cautious wingspan estimate of 3.0–3.5 m seems most plausible for *Gobiazhdarcho tsogtbaatari*.

Regarding the holotype and only known specimen of *Tsogtopteryx* *mongoliensis*, the only preserved element available is unfortunately a partial CVI, greatly limiting comparisons. Still, it is notable that MPC−Nd 100/303 represents a very small specimen. The prezygapophyseal width is of 25 mm, what corresponds to 38% the same value in *Q. lawsoni* specimen TMM 42180-19 (~60 mm), suggesting a wingspan of ~1.6–1.9 m for MPC−Nd 100/303. Similarly, cotylar width is 15 mm, corresponding to ~40% the same value in *Q. lawsoni* TMM 42180-19 (~37 mm), suggesting a wingspan of ~1.7–2 m for MPC−Nd 100/303. Therefore, an estimate of <2 m seems plausible for *Tsogtopteryx* *mongoliensis*. As explored above, the Burkhant specimen is probably very close to reaching osteological maturity, while the Bayshin Tsav can be regarded as osteologically mature.

1. ***Strict consensus tree (complete)***

***
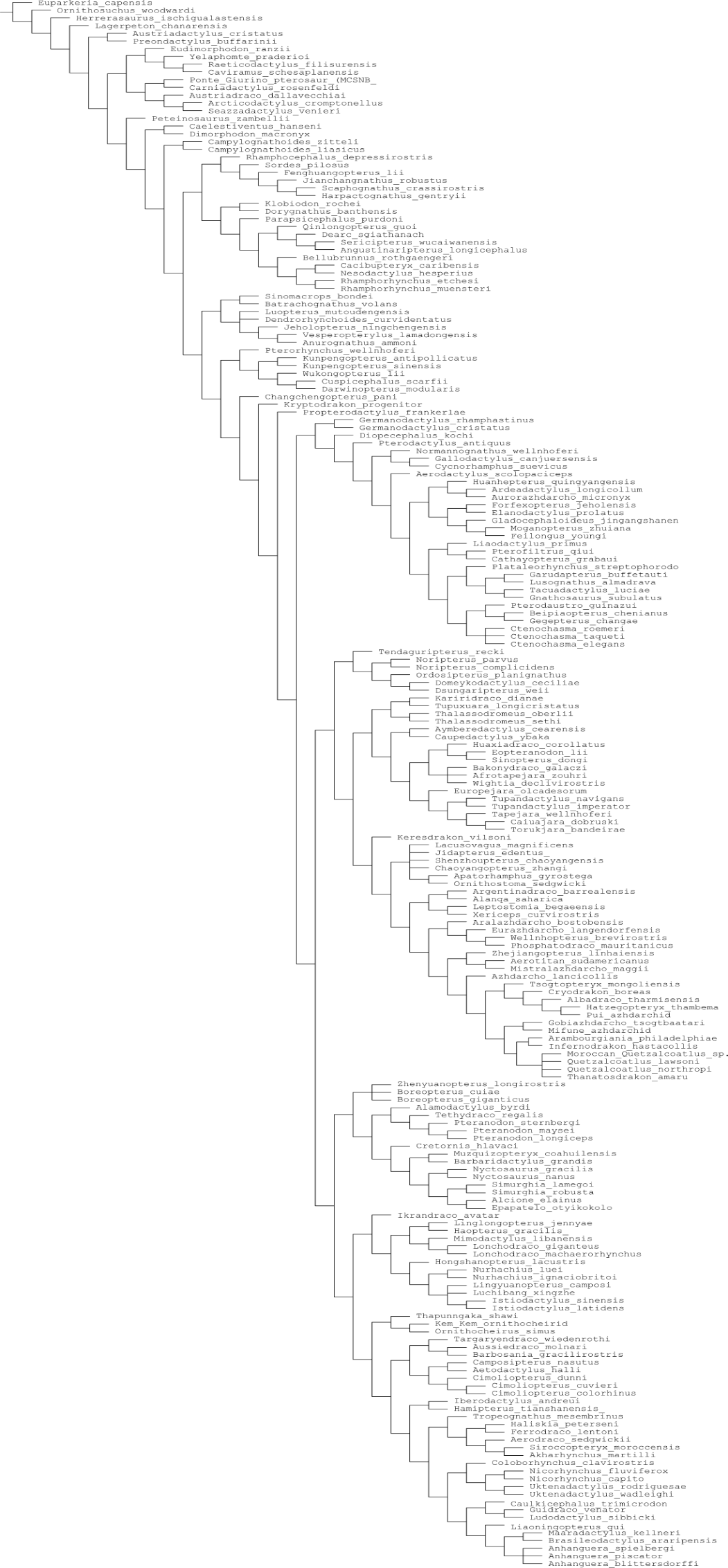
***

**Supplementary References**

Andres, B., & Langston Jr, W. (2021). Morphology and taxonomy of *Quetzalcoatlus* Lawson 1975 (Pterodactyloidea: Azhdarchoidea). *Journal of Vertebrate Paleontology*, *41*(sup1), 46-202.

Cai, Z. and Wei, F. 1994. On a new pterosaur (*Zhejiangopterus linhaiensis* gen. et sp. nov.) from Upper Cretaceous in Linhai, Zhejiang, China. *Vertebrata Palasiatica* 32: 181–194

Hone, D. W., Habib, M. B., & Therrien, F. (2019). *Cryodrakon boreas*, gen. et sp. nov., a Late Cretaceous Canadian azhdarchid pterosaur. *Journal of Vertebrate Paleontology*, *39*(3), e1649681.

Naish, D., & Witton, M. P. (2017). Neck biomechanics indicate that giant Transylvanian azhdarchid pterosaurs were short-necked arch predators. *PeerJ*, *5*, e2908.

Pêgas, R. V., Holgado, B., David, L. D. O., Baiano, M. A., & Costa, F. R. (2021). On the pterosaur *Aerotitan sudamericanus* (Neuquén Basin, Upper Cretaceous of Argentina), with comments on azhdarchoid phylogeny and jaw anatomy. *Cretaceous Research*, *129*, 104998.

Vremir, M., Kellner, A. W., Naish, D., & Dyke, G. J. (2013). A new azhdarchid pterosaur from the Late Cretaceous of the Transylvanian Basin, Romania: implications for azhdarchid diversity and distribution. *PLoS One*, *8*(1), e54268.

Vremir, M., Witton, M., Naish, D., Dyke, G., Brusatte, S. L., Norell, M., & Totoianu, R. (2015). A medium-sized robust-necked azhdarchid pterosaur (Pterodactyloidea: Azhdarchidae) from the Maastrichtian of Pui (Haţeg Basin, Transylvania, Romania). *American Museum Novitates*, *2015*(3827), 1-16.

Vullo, R., Garcia, G., Godefroit, P., Cincotta, A., & Valentin, X. (2018). *Mistralazhdarcho maggii*, gen. et sp. nov., a new azhdarchid pterosaur from the Upper Cretaceous of southeastern France. *Journal of Vertebrate Paleontology*, *38*(4), 1-16.
